# Artificial intelligence - based ultrasound elastography for disease evaluation - a narrative review

**DOI:** 10.3389/fonc.2023.1197447

**Published:** 2023-06-02

**Authors:** Xian-Ya Zhang, Qi Wei, Ge-Ge Wu, Qi Tang, Xiao-Fang Pan, Gong-Quan Chen, Di Zhang, Christoph F. Dietrich, Xin-Wu Cui

**Affiliations:** ^1^ Department of Medical Ultrasound, Tongji Hospital, Tongji Medical College, Huazhong University of Science and Technology, Wuhan, China; ^2^ Department of Ultrasonography, The First Hospital of Changsha, Changsha, China; ^3^ Health Medical Department, Dalian Municipal Central Hospital, Dalian, China; ^4^ Department of Medical Ultrasound, Minda Hospital of Hubei Minzu University, Enshi, China; ^5^ Department of Medical Ultrasound, The First Affiliated Hospital of Anhui Medical University, Hefei, China; ^6^ Department of Internal Medicine, Hirslanden Clinic, Bern, Switzerland

**Keywords:** ultrasound, elastography, artificial intelligence, machine learning, deep learning, radiomics

## Abstract

Ultrasound elastography (USE) provides complementary information of tissue stiffness and elasticity to conventional ultrasound imaging. It is noninvasive and free of radiation, and has become a valuable tool to improve diagnostic performance with conventional ultrasound imaging. However, the diagnostic accuracy will be reduced due to high operator-dependence and intra- and inter-observer variability in visual observations of radiologists. Artificial intelligence (AI) has great potential to perform automatic medical image analysis tasks to provide a more objective, accurate and intelligent diagnosis. More recently, the enhanced diagnostic performance of AI applied to USE have been demonstrated for various disease evaluations. This review provides an overview of the basic concepts of USE and AI techniques for clinical radiologists and then introduces the applications of AI in USE imaging that focus on the following anatomical sites: liver, breast, thyroid and other organs for lesion detection and segmentation, machine learning (ML) - assisted classification and prognosis prediction. In addition, the existing challenges and future trends of AI in USE are also discussed.

## Introduction

Hardness or stiffness is an important biomarker of abnormal tissue. Changes in tissue hardness are often accompanied by common disease progression (1). It is also well known that cancerous tissue tends to be stiffer than benign and normal tissues (2). Ultrasound elastography (USE) is an emerging imaging technology sensitive to tissue stiffness. By adding the tissue stiffness as another measurement characteristic to the conventional ultrasound imaging system, USE can provide added power for improving the diagnostic performance in various diseases. USE has been gradually applied to the evaluation of diseases in some superficial organs, where tissue stiffness is closely related to the specific pathological process, such as characterizing breast masses (3) and thyroid nodules (4), assessing liver fibrosis (5) and detecting prostate lesions (6).

However, due to the great intra- and inter-observer variabilities and the instable diagnostic performance or even low accuracy with the manual interpretation of inexperienced radiologists, the analysis of medical images is challenging, especially for USE images in which the boundary of a lesion is usually implicit (7). The limitations mainly lie in the difficulty of identifying optimal stiffness cutoff values, the variability of the region of interest (ROI) selection and the lack of an image quality check (8). Besides, a wide disparity in diagnostic performance has been reported (9).

Artificial intelligence (AI) has been recognized as the Fourth Industrial Revolution because it is reshaping multiple fields worldwide, ranging from facial recognition to self-driving vehicles or natural language processing (10). Interpretating medical images is inherently a data processing step in which AI can be applied in the medical domain (11). Moreover, the availability of novel AI techniques, emerging imaging techniques and massive imaging datasets have made medical imaging a research field of AI (12). As a subset of AI, the rapid development of machine learning (ML) approaches, especially the advanced deep learning (DL) architectures, offer great potential to perform automatic medical image analysis tasks, such as segmentation, detection and classification (13).

Researches have already shown the significant value of ML- or DL-based analysis using conventional gray scale ultrasound images for diseases evaluation (14–17). As USE has gradually been used as a complement to conventional ultrasound by providing information on tissue elasticity, there is a growing trend in applications of AI-based USE images. By removing variability between examiners, these developed models have extensively enabled the accuracies of image interpretation and allowed the disease diagnosis go much further.

Although reference (18) was the first review on DL methods in the USE imaging, this article mainly reviews novel DL architectures applied to USE from an engineering perspective. Reference (19) covers a limited number of articles about the ML models applied to USE for breast tumor classification. Therefore, to our knowledge, there is no literature that provides radiologists with a comprehensive and clinically-oriented review of AI applied to USE imaging in a more easily-readable manner. In this review, a brief overview of USE and AI techniques (including ML, DL and radiomics) is provided. Then, the applications of AI-based USE for disease evaluation in several anatomical organs in the order of automatic analysis tasks are introduced. Finally, the existing challenges and future trends with the application of AI based on USE are discussed.

## Overview of ultrasound elastography

**Figure 1:**
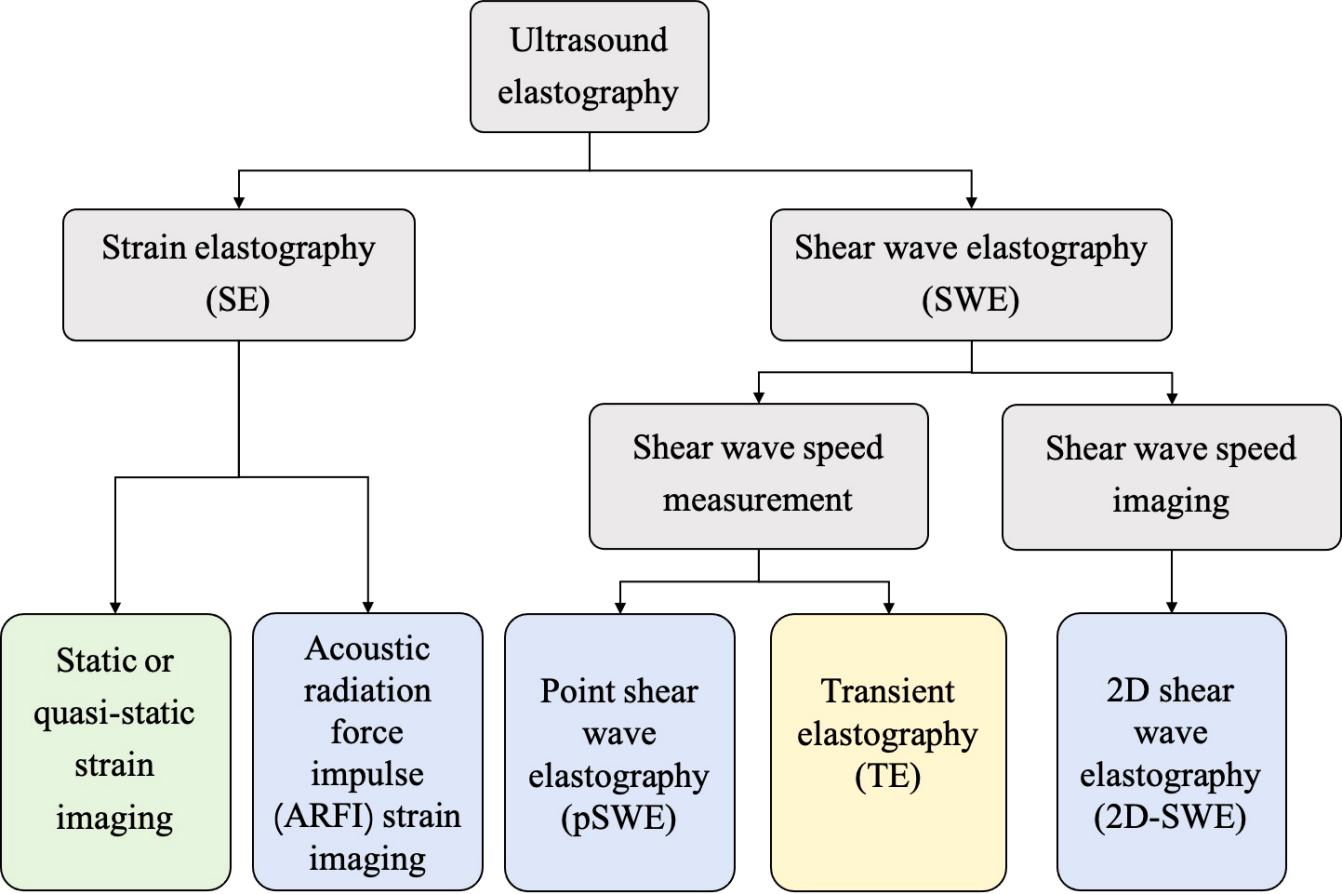
The category of ultrasound elastography (USE) technique according to the excitation methods, including external compression or internal physiologic motion (green), acoustic radiation force impulse (blue), and mechanical vibrating (yellow).

In general, the USE technique can be classified into strain elastography (SE) applying constant stress and shear wave elastography (SWE) applying time-varying force. **Figure 1** shows the category of the USE technique according to excitation methods. All of the approaches are based on the three-phase methodology: (a) tissue is compressed by static stress or shear wave propagation; (b) the displacements in tissues are tracked by ultrasound; and (c) tissue elasticity is estimated quantitatively or qualitatively from the measured displacements. Moreover, a physical property named as Young’s modulus (*E*) is calculated to estimate the tissue stiffness. Harder lesions have smaller deformations, lower strains and higher *E* values (1).

### Strain elastography

SE was the first elastography imaging system introduced in the 1990s and can be divided into two methods (20): (a) static or quasi-static strain imaging: the operator manually compresses the tissue with an ultrasound transducer; and (b) acoustic radiation force impulse (ARFI) strain imaging: a short-duration high-intensity acoustic “pushing” pulse called ARFI can be used to displace the tissue (21).

According to Hooke’s law, *E* in strain imaging can be calculated by the equation *E=σ/ϵ*, where *σ* is the externally applied stress and *ϵ* is the strain, which equals the displacement (21). Due to the unknown applied stress to the tissue, the strain imaging system cannot provide the qualitative value of *E*. Therefore, in clinical practice, strain ratio (SR) is an often-used semiquantitative measurement. SR can be calculated as the ratio between the strain in the normal reference region and the strain in the region of interest. SR>1 indicates that the deformation of the target lesion is less than that of the normal reference tissue, indicating lower strain and greater hardness (1). There are some other common parameters: elasticity scores (ES) or grading systems, fat-to-lesion SR and elastography-to-B-mode size ratio (1).

### Shear wave elastography

SWE, which is more quantitative and reproducible than SE, can be classified into two methods: (a) transient elastography: The first commercial SWE system Fibroscan™ is based on transient elastography, which is widely used to estimate liver fibrosis (5); (b) point shear wave elastography and 2D-SWE: ARFI is used as the external excitation.

Moreover, in contrast to nonimaging USE methods (transient elastography and point shear wave elastography), 2D-SWE is an emerging technology that can measure shear wave velocity (SWV) or *E* in real time and generate quantitative elastograms (21). The semitransparent color elastogram is usually overlayed on the corresponding B-mode sonogram, with red usually representing hard tissue and blue representing soft tissue. The SWV and *E* are related to the colors in the color bar along the image (22). The system will report *E* by using the equation *E=3ρc_s_^2,^* in which *ρ* represents the tissue density and *c_s_* represents the SWV (1). SWV is higher in hard tissue and lower in soft tissue.

## Overview of artificial intelligence

### Machine learning

As a branch of AI, ML enables the creation of algorithms that are able to learn from data and make predictions, thus enabling computers to learn like humans (23). It is an interdisciplinary field involving computer science, statistics and a variety of other disciplines concerning automatic improvement (24).

**Figure 2:**
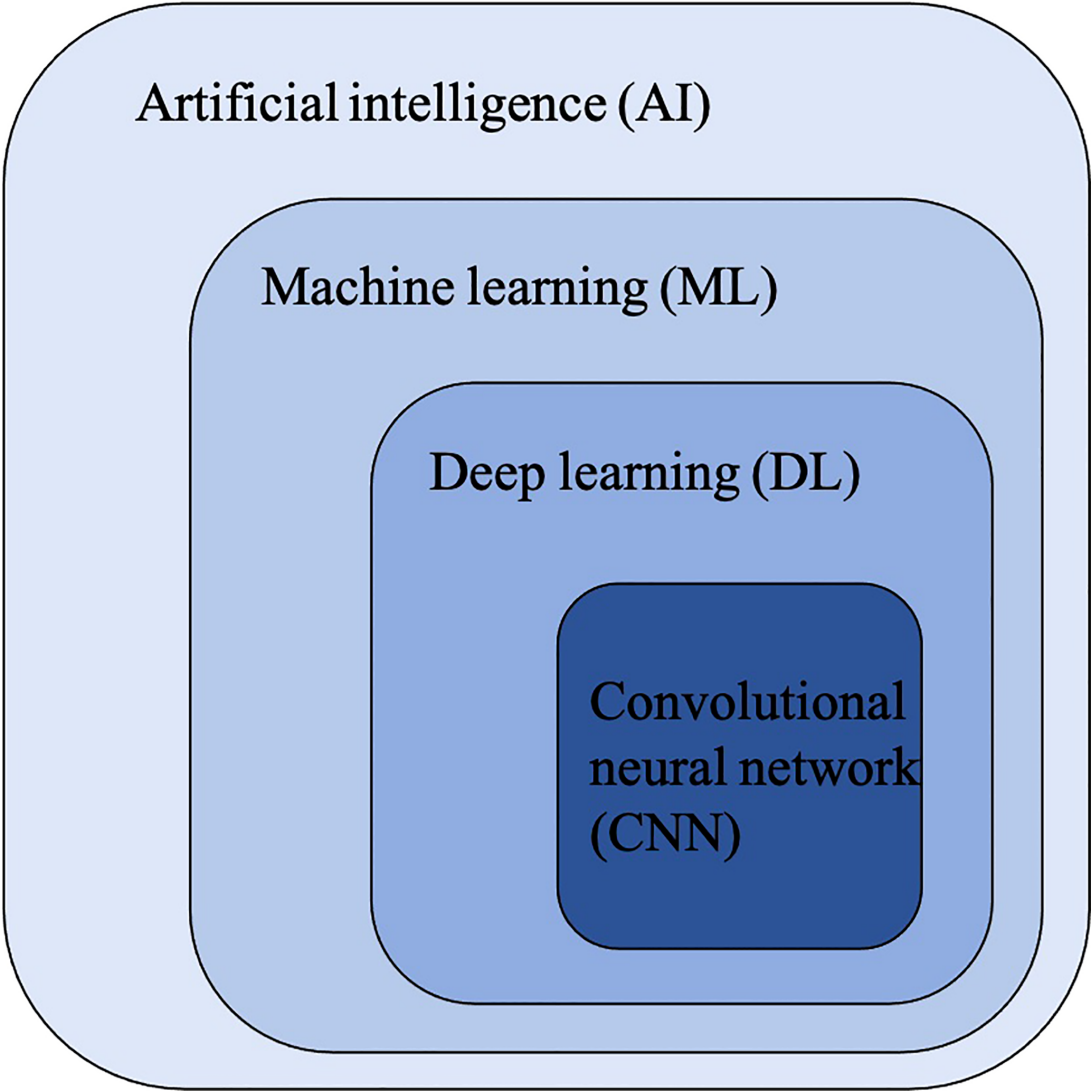
The relationship between artificial intelligence (AI), machine learning (ML), deep learning (DL) and convolutional neural networks (CNNs).

According to the types of labels utilized in the training dataset, ML techniques can be broadly classified by supervised, unsupervised and reinforcement learning (25) (**Figure 2**). Most ML algorithms related to radiology are supervised (11), which requires instances labeled with the desired classification outputs, named the “ground truth”. Examples of such algorithms include artificial neural networks (ANNs), support vector machine (SVM), random forest (RF), logistic regression, Naïve Bayes classifier, decision tree and K-nearest neighbor. In contrast, unsupervised learning is an algorithm in which instances are unlabeled and clusters of data need to be identified (26). Examples of such algorithms are K-means, fuzzy C-means clustering and Markov random fields. Since acquiring well-labeled databases is time-consuming, reinforcement learning, as a hybrid of supervised and unsupervised learning, uses less detailed information to train a model (27). It acquires data by learning from dynamic environment interaction (the computer will receive positive or negative reinforcement feedback) without being explicitly taught. The overview of ML strategies and their related algorithms and applications used in USE imaging has been presented in **Table 1**.

**Table 1:** Overview of machine learning strategies and their related algorithms and popular deep learning architectures.

Types of learning	Types of algorithms	Characteristic	
Supervised learning	Support vector machine	The algorithm consists of a multidimensional hyperplane to obtain the optimal solution for classification using statistical methods. It is useful for taking a large number of features and discriminating inputs into one of two classes, which can be then applied to classification and regression tasks.	
	Logistic regression	The algorithm is a regression method which takes the feature as the argument and takes the category as the dependent variable. If the data is nonlinearly separable, the performance of linear classifier will be unsatisfactory.	
	Naïve Bayes classifier	The algorithm is a simple probabilistic classification method based on Bayes theorem and assumes that all features are independent of each other. It estimates parameters using the method of maximum likelihood and often leads to more robust results when there are relatively small datasets.	
	Random forest	The algorithm is a classifier with multiple decision trees, and its output is determined by the common value of the categories output from the individual trees. It shows relatively good performance for ensemble-based classifications while overfitting has been observed for noisy data.	
	Decision tree	The algorithm uses a flowchart-like tree model with multiple branch nodes to determine a target value from the input. It is useful to perform classification (classification trees) but is inadequate for regression and continuous value prediction tasks.	
	K-nearest neighbor classifier	The algorithm is an instance-based classifier where the classification of an unknown sample is performed by relating the unknown to a known sample according to some distance or similarity criteria. It is used both for classification and regression tasks but it’s time-consuming and computationally expensive.	
	Artificial neural network	The algorithm consists of three components, including the input layer, hidden layer, and output layer which simulates neurons and classifies new individuals through learning and training processes. It can map complex nonlinear relationships between dependent and independent variables but requires a large well-annotated dataset to achieve good performance.	
Unsupervised learning	K-means	The algorithm measures the distance between each pair of data points and the specified clustering centroid to classify the data, and optimizes the allocation by comparing intra- and inter-family data point distances. It can process large datasets while umber of clusters must be defined.	
	Clustering methods	The algorithm aims to achieve the goal of reducing the need of large amount of data and performing classification by optimally finding similarities or clusters from data.	
Reinforcement learning	Not applicable	Data labels are acquired by learning from dynamic environment interaction (the computer will receive positive or negative reinforcement feedback) without being explicitly taught.	
Popular DL architetcures			
AlexNet		one of the first high performance classification architecture which is characterized by using ReLUs activation function, dropouts and data augmentation	
GoogleNet		includes inception structures in which input data are processed with different functions and the results are concatenated; good at image classification	
VGGNet		uses only several deeper layers and smaller filter kernels	
ResNet		uses skip structures with which input data are added to processed data, helping the network learn residuals and fine details	
R-CNN		a tryout of DL methods on detection tasks which is a two-stage network by fine-tuning on the model trained in the classification task	
YOLO		a one-stage network which is fast and simplified and can help perform real-time object detection and classification	
U-Net		consists of a contracting path on the left side (encoder) and an expansive path on the right side (decoder) for fast and precise biomedical image segmentations	
GANs		consists of one generator and one classifier which can help generate synthetic medical images to train deeper architectures	

In the field of radiology, ML algorithms often start with a set of available inputs (the image data, for example) and desired outputs (the classification of malignant and benign tumors, for example) (11). The input datasets are usually split into two sets: training and validation datasets. The training dataset serves to find the optimal weights and fit the model, while the validation dataset is used to optimize the parameters. After a model is developed, there is often an urgent need for an independent external test dataset to evaluate the performance and generalizability of the developed model (23).

### Deep learning

The availability of large-scale labeled datasets, faster algorithms and more powerful parallel computing hardware, such as Graphics Processing Unit, have enabled the fast application of DL (28). DL methods enable to solve problems that have resisted the best attempts of the AI community for many years, since the use of large numbers of layers allow the improved universal approximation properties and the more features to be learned from the data with multiple levels of hierarchy and abstraction (29).

**Figure 3:**
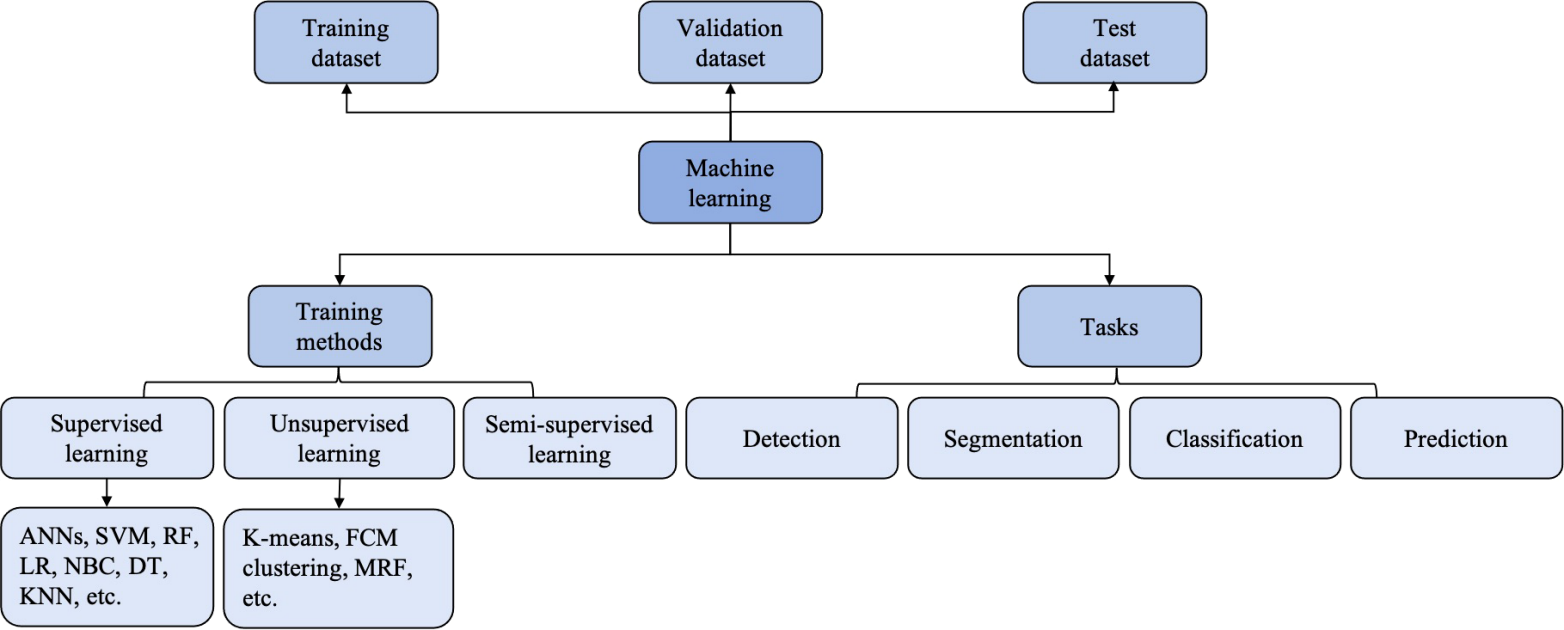
The classification and computer vision task of machine learning (ML). ANNs, artificial neural networks; DT, deciosin tree; FCM clustering, fuzzy C-means clustering; KNN, K-nearest neighbor; LR, logiastic regression; MRF, Markov random fields; NBC, Naïve Bayes classifier; RF, random forest; SVM, support vector machine.

**Figure 4:**
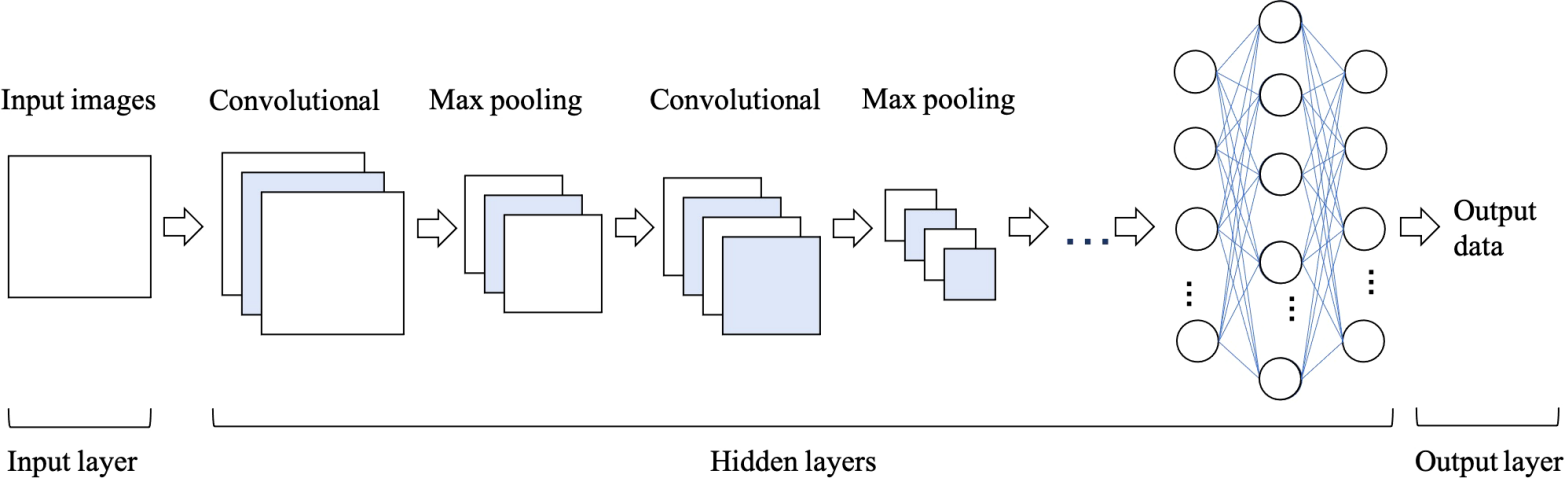
The architecture of convolutional neural network (CNN), which is formed of one input layer, multiple hidden layers and one output layer. Convolutional and max pooling layers can be stacked alternately until the network is deep enough to acquire optimal features of the images that are salient for classification task.

Convolutional Neural Networks (CNNs) have been considered as the state-of-art algorithms of DL methods in the field of radiology for computer vision tasks such as segmentation (30), detection (31, 32) and classification (33). There are four key ideas behind CNNs: local connections, shared weights, pooling and the use of many layers, resulting in the improved accuracy and efficiency of the whole system (29). The relationship between AI, ML, DL and CNNs is shown in **Figure 3**. CNNs are formed by a stack of one input layer, one output layer and multiple hidden layers, which consist of convolutional layers, pooling layers and fully-connected layers (34) (**Figure 4**). With convolution and pooling applied repeatedly, fully-connected layers are used to make classification or predictions (35). The combinations of layers are various and some deep neural network architectures have been successfully utilized in image analysis, such as GoogleNet (36), AlexNet (37), VGGNet (38) and ResNet (39).

### Transfer learning

Transfer learning (TL) strategies have recently been utilized in the medical world to avoid the overfitting problems caused by the lack of data. Within the TL method, knowledge can be shared and transferred between different tasks (40). The workflow involves two steps: pretrained on a large dataset (the ImageNet, for example) and fine-tuning on the target dataset (the limited ultrasound images, for example). In other words, by fine-tuning the DL architecture, the knowledge learned from one dataset can be transferred to another dataset obtained from another center.

### Radiomics

Radiomics is defined as quantitative mapping, namely, to extract and analyze vast arrays of high-dimensional medical image features that are related to the prediction targets (41). Radiomics features, such as intensity, shape, texture or wavelet, reflect the underlying pathophysiology and provide the information on tumor phenotype and microenvironment (42). These features can be used solely or in combination with other relevant data sources (such as clinical reports, laboratory tests or genomic data) for robust evidence-based decision support to facilitate prediction, diagnosis, prognosis, or monitoring (41).

Radiomics features can be scored semantically by experienced radiologists, or can be mathematically computer-calculated to describe the size, shape and textures of the ROI, or can be created by DL algorithms (43). The calculated features are subsequently analyzed usually using traditional ML methods to build predictive models (44). Researches have already shown the capacity of radiomics analyses to increase diagnostic, prognostic, and predictive power (42).

## Applications of AI in USE imaging

The primary automatic analysis tasks of AI-based USE models involve (13) (a) classification: to predict the target class labels of an image (to classify breast tumors as benign or malignant, for example); (b) detection: to predict the location of focal lesions, which is a prerequisite step for radiologists to characterize lesions; (c) segmentation: to distinguish the suspicious lesions from the surrounding normal tissues, that is, to acquire the ROI; and (d): prediction: to predict the status of disease or events that may happen.

The early developed AI-based models follow the traditional method: computing the handcrafted features, applying the feature selection algorithm to reduce the features in dimension, and training a classifier to acquire the best results (28). Overall, feature extraction and selection are the most important steps, helping to achieve the best diagnostic results (45).

**Figure 5:**
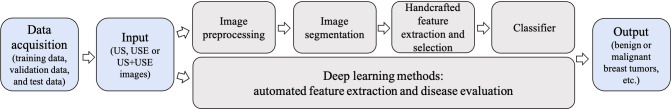
Conventional machine learning (ML)-based ultrasound elastography (USE) models vs deep learning (DL)-based USE models. Conventional ML-based USE models depend on carefully handcrafted features, while DL allows learning an end-to-end mapping from the input to the output.

Compared to conventional ML methods, the key aspect of DL algorithms is that these layers of features are not handcrafted with human expertise: they are automatically learned from data using a general-purpose learning procedure (29). It allows learning an end-to-end mapping from the input to the output, that is, the image-to-classification methods (13). In traditional ML methods, the accurate segmentation and choice of expert-designed features are keys to success. These limitations can be overcome by DL approaches, because these algorithms can identify the regions of the image that are most associated with the outcome by training themselves and can identify the features of the region that informed decision by multiple layers (43). The comparison of the traditional ML-based models and DL-based models is shown in **Figure 5**.

The more influential literature on the applications of AI in USE images are subsequently summarized in terms of anatomical sites. USE is prone to be performed in most of the organs, including breast, thyroid, liver and so on.

## Liver disease evaluation

Many etiologies of chronic liver diseases (CLDs) follow a common pathway to liver fibrosis, cirrhosis and hepatocellular carcinoma (HCC). HCC is the third highest cause of cancer mortality worldwide (46). Liver biopsy has traditionally served as the gold standard for staging liver fibrosis in CLD and diagnosing HCC. However, it is invasive and has some limitations, such as sampling error and postoperative complications (47). Compared to standard ultrasound, USE has become a popular non-invasive technique by providing additional information regarding liver tissue stiffness. AI-based SE models can help improve the diagnostic accuracy while reducing the unnecessary biopsies for either staging liver fibrosis in CLD patients or differentiating focal liver lesions (FLLs) from benign to malignant.

### Staging liver fibrosis

Liver fibrosis is a principal factor in the development of CLD. Precise assessment of the amount and progression of liver fibrosis is of great value for the management and prognosis of CLD patients. Liver stiffness measured from USE has been regarded as the first-line noninvasive method; thus, AI-based USE models is proposed to reduce variability in stiffness measurements and to improve the diagnostic accuracy of liver fibrosis evaluation.

Earlier studies have extracted a series of hand-engineered features and then fed into various ML classifiers for predicting the different liver fibrosis levels. Textural features, liver stiffness and SWV have been proven to be the discriminant features, with ANN, SVM and RF being the most used classifiers (48–54).

More currently, AI with DL methods is expected to directly operate on the input images and reveal information that human experts cannot recognize. The better diagnostic performance of CNN than conventional ML classifiers for staging liver fibrosis has been demonstrated in the study of Brattain and coworkers (55). To obtain more standard SWE measurements and to detect significant fibrosis in patients with nonalcoholic fatty liver disease, they developed an automated framework called “SWE-Assist”. The model could automatically check the quality of SWE images, select a ROI, and classify the ROI with RF, SVM classifiers and CNNs. The CNN architecture yielded the largest classification improvement with an AUC of 0.89 in a large dataset of 3392 SWE images. A new method called DL radiomics of elastography (DLRE) has been proposed by Wang et al., which was expected to extract more radiomics features (56). In 398 patients with 1990 images from 12 hospitals, DLRE reached AUCs of 0.97 for F4, 0.98 for ≥ F3 and 0.85 for ≥ F2, which were significantly better than LSM and biomarkers. They also found that the model generated better performance as more images were acquired. As the DLRE model in Wang et al. showed limited accuracy in assessing ≥ F2, Lu et al. developed a new model named DLRE 2.0 based on the previous model by using the TL strategy for a more accurate evaluation of ≥ F2 liver fibrosis (57). They also developed three other DLRE-based models by gradually adding the tissue texture of the liver capsule and the liver parenchyma and serological results to DLRE 2.0. For evaluating ≥ F2, the performance of DLRE2.0 was significantly better than the previous DLRE model (AUCs: 0.84 vs 0.92, p < 0.05). However, no significant improvement was shown when adding more information. By using the TL strategy for classifying liver fibrosis, Xue et al. pretrained the InceptionV3 network on ImageNet to analyze multimodal BMUS and SWE images, generating higher diagnostic accuracy than non-TL with significantly higher AUCs (0.950, 0.932, and 0.930 for classifying F4, ≥ F3, and ≥ F2, respectively**)** (58). Furthermore, the model based on the multi-modal images yielded the highest diagnosis accuracy, outperformed the single-modality, LSM, and biomarkers.

How to define a reliable SWE image, especially a temporally stable SWE color box, is one of the limitations of USE. Gatos et al. tried to identify the temporally stable regions across SWE frames and to evaluate the impact of temporal stability for classifying various CLD combinations by means of a pretrained CNN scheme (59). The stability masked SWE images showed improved diagnostic performance compared to the unmasked ones. Therefore, in their study in 2020 (60), full and temporally stable masked SWE images were separately fed into GoogleNet, AlexNet, VGG16, ResNet50 and DenseNet201 with or without augmentation. All networks achieved maximum mean accuracies ranging from 87.2%–97.4% and AUCs ranging from 0.979–0.990.

### Classification and evaluation of focal liver lesions

Early detection and precise diagnosis of HCC are crucial for treatment selection and patient prognosis, making the differentiation of FLLs an important task. Since structural alterations can be reflected by changes in tissue stiffness, USE, especially SWE, has been widely used for differentiating benign from malignant FLLs (61, 62).

For different diagnostic purposes (all including FLL classification), radiomics approaches have been proposed with an emphasis on the extraction of high-dimensional features from different modality ultrasound images and the integration of the high-dimensional features with the low-dimensional data, such as expert-designed SWE parameters or serological clinical data (63, 64). Specifically, to classify FLLs, Wang et al. established two radiomics models: the ultrasomics score (based on radiomics features only) and the combined score (based on radiomics features and quantitvative SWE measurements) (63). With 1044 features extracted by ultrasomics, they found that the combined score had the best performance, yielding an AUC of 0.92. Chronic hepatitis B infection is the main risk factor for HCC in China. Early and accurate prediction of HCC occurrence in chronic hepatitis B patients is of great benefit for individual treatment and prognosis. Jin et al. established different HCC prediction models by combining high-throughput radiomics features using DL radiomics with serological and clinical information (64). Finally, the model including SWE, BMUS radiomics features, sex and age showed the best prediction performance (AUCs: 0.981, 0.942 and 0.900 in training, validation and testing cohorts for predicting 5-year prognosis of HCC).

The prediction of malignant subtype and clinical prognosis are also important for the decision-making and treatment of HCC patients. Yao et al. have demonstrated the value of the radiomics analysis system based on multi-modal ultrasound images (BMUS, SWE and SWV imaging) for benign and malignant classification, malignant subtyping, programmed cell death protein 1 prediction, Ki-67 prediction, and microvascular invasion prediction, with the AUCs ranging from 0.94-0.98 (65). The more detailed performance of AI based on USE for the evaluation of liver diseases is summarized in **Table 2**.

**Table 2:** The performance summary of artificial intelligence applied to ultrasound elastography in the evaluation of liver diseases.

Year	Authors	No. of patients	Device	Modality	Training targets	Method	Features	Performance
2011	Stoean et al. (49)	722	Fibroscan	SWE	Stage fibrosis in CHC	ESVM, GA	LSM, hematological, biochemical indicators	Mean accuracy: 77.31%
2013	Fujimoto et al. (48)	310	Hitachi	SE	Stage fibrosis in CHC	LR	Texture features	Correlation coefficient r=0.68
2015	Procopet et al. (50)	202	Fibroscan	SWE	Diagnosis cirrhosis, CSPH	ANN	LSM, serum tests	Accuracy: 86.3% for cirrhosis; 84% for CSPH; 81.8% for esophageal varices
2016	Gatos et al. (51)	85	SuperSonic	SWE	Classify healthy from CLD patients	inverse mapping technique, SRA, SVM	Texture features	AUC: 0.85 Accuracy: 87.0% Sensitivity: 83.3% Specificity: 89.1%
2017	Gatos et al. (52)	126	SuperSonic	SWE	Classify healthy from CLD patients	inverse mapping technique, stiffness value-clustering, SRA, SVM	Quantitative features	AUC: 0.87 Accuracy: 87.3% Sensitivity: 93.5% Specificity: 81.2%
2017	Chen et al. (54)	513	Hitachi	SE	Stage fibrosis in CHB	SVM, NBC, RF, KNN	Quantitative features, texture features	Accuracies: 82.87% for ≥ F1; 81.18% for ≥ F2; 88.09% for ≥ F3; 91.25% for F4 (RF classifier)
2018	Brattain et al. (55)	328	SuperSonic	SWE	Detect ≥F2 fibrosis in NAFLD	RF, SVM, CNN		AUC of CNN: 0.89
2018	Yao et al. (65)	177	Canon	BMUS, SWE, SWV imaging	Classify FLLs; pathologic diagnosis and prognostic prediction of HCC	SRT, SVM		AUCs: 0.94 for FLLs classification; 0.97 for malignant subtyping; 0.97 for PD-1 prediction; 0.94 for Ki-67prediction; 0.98 for MVI prediction
2019	Wang et al. (56)	398	SuperSonic	SWE	Stage fibrosis in CHB	DLRE		AUCs of DLRE: 0.97 for F4; 0.98 for ≥F3; 0.85 for ≥F2
2019	Gatos et al. (59)	200	SuperSonic	SWE	Diagnosis CLD	Inverse mapping technique, DWT, FCM clustering, CNN		AUCs: 0.93-0.99 Accuracy: 82.5%-95.5% (Masked images)
2020	Xue et al. (58)	466	SuperSonic	BMUS, SWE	Stage fibrosis in CLD	CNN, TL		AUCs: 0.950 for F4; 0.932 for ≥F3; 0.930 for ≥F2 (Multimodal images)
2020	Kagadis et al. (60)	200	SuperSonic	SWE	Diagnosis CLD	CNNs		AUCs: 0.979-0.990 Accuracy: 87.2%-97.4% (All five networks)
2021	Durot et al. (53)	204	Siemens, Philips	SWE, MRE	Classify non-significant from significant fibrosis	LR, NBC, QDA, SVM	SWV	AUC: 0.962 Accuracy: 90.2% Sensitivity: 81.3% Specificity: 94.7% (pSWE-based SVM) AUC: 0.987 Accuracy: 96.7% Sensitivity: 89.5 Specificity: 100.0% (2D SWE-based SVM)
2021	Lu et al. (57)	807	SuperSonic	SWE	Detect ≥F2 fibrosis in CLD	DLRE, TL		AUCs: 0.91 for ≥F2; 0.97 for cirrhosis
2021	Wang et al. (63)	169	SuperSonic	SWE	Classify FLLs	Ultrasomics-Platform, SVM		AUC: 0.94 Sensitivity: 92.59% Specificity: 87.50%
2021	Jin et al. (64)	434	SuperSonic	BMUS, SWE	Predict HCC in CHB	DLRE		AUCs: 0.981in training cohort

CHB, chronic hepatic B; CHC, chronic hepatic C; CLD, chronic liver diseases; CSPH, clinically significant portal hypertension; DLRE, deep learning radiomics of elastography; DWT, dyadic wavelet transformation; ESVM, the evolutionary-driven support vector machines; FLL, focal live lesion; HCC, hepatocellular carcinoma; LSM, liver stiffness measurement; MRE, MR elastography; NAFLD, non-alcoholic fatty liver disease; QDA, quadratic discriminant analysis; SRA, stepwise regression analysis; SRT, sparse representation theory; SWE, shear wave elastography; SWV, shear wave velocity.

## Breast mass evaluation

Breast cancer is the most common cancer diagnosed in women (66). Accurate breast mass evaluations, including segmentation and detection, differentiation of benign from malignant breast masses, and prediction of axillary lymph node (ALN) status and treatment response, can lead to individualized treatment and favorable prognosis. AI methods applied to USE are expected to provide a more objective and reproducible evaluation for breast masses.

### Segmentation and delineation of breast masses

Accurate delineation of breast masses on ultrasound images is an indispensable first step for image interpretation. Manual segmentation of breast masses is labor intensive and time-consuming (67). The various artifacts, uneven intensities and blurred boundaries in USE images have made automated segmentation of breast mass still a tough task (68). Sergiu and coworkers developed a probabilistic model for every pixel derived by a video sequence, followed by a Deterministic Annealing Expectation Maximization method used for automatic image segmentation of breast elastographic images (69). Only an error of 5% on the phantom test images was provided.

### Classification and diagnosis of breast masses

Early detection and treatment of breast cancer can significantly decrease mortality. Therefore, the differential diagnosis of benign and malignant breast masses is of great value in breast mass evaluation. Although the American College of Radiology Breast Imaging Reporting and Data System (ACR BI-RADS) can provide a standardized and systemic interpretation of breast ultrasound, the problems of inter- and intra-observer variability can be enormously addressed by AI solutions (70).

Early AI-based USE models for breast mass classification focused on the extraction of discriminant features and the utilization of various classifiers. The elasticity indices, such as SR and quantitative tissue elasticity, as well as texture features have served as the most commonly used handcrafted features (71–77). ANN and SVM classifiers are the most commonly used ML algorithms, all yielding satisfying performance. The fused ultrasound B-mode and elastographic features, have all been proven to generate better results than single-modal imaging (71, 72, 75, 77–83). It is also proved that the elasticity features along the rim surrounding the lesion is valuable for the classification of breast masses (84).

However, it is difficult to extract human-crafted features from USE images since they often contain irrelevant patterns (28), and the classification performance is greatly influenced by the selection of particular features. Recently, DL algorithms, especially CNNs, have made great strides in establishing AI-based USE models for breast mass classification (85–88). Zhang et al. developed a two-layer DL architecture based on SWE, comprising a pointwise gated Boltzmann machine and a restricted Boltzmann machine (85). The pointwise gated Boltzmann machine, restricted Boltzmann machine and SVM classifiers were used for automated feature learning, distinct representation learning and classification of breast tumors, respectively. Compared with the handcrafted statistical features, an accuracy of 93.4%, a sensitivity of 88.6%, a specificity of 97.1%, and an AUC of 0.947 were achieved. Fujioka et al. developed a DL model based on SWE images using six CNN architectures with different epochs for breast mass classification (86). The developed DL model reached a mean AUC of 0.870, showing equal or better diagnostic performance compared with the radiologists who analyzed the images using the 5-point visual color assessment and the mean elasticity value. Li et al. found that the CNN-based model using dual modal ultrasound images could provide a more pronounced improvement in diagnostic performance for inexperienced radiologists, with the AUC increasing from 0.794 to 0.830 (87).

Radiomics can provide automated quantification of high-throughput image features. It is expected to reveal disease characteristics that are invisible to the naked eye (42). Generally, the proposed ML-based radiomics models focused on the extraction of low- and high-order features and the utilization of various feature selection algorithms (89–91). The radiomics features involve shape, intensity, texture or wavelet features from different modality ultrasound images, such as SE (89), SWE (90), contrast-enhanced ultrasound (CEUS) (91) and B-mode ultrasound (BMUS) (90, 91). The frequently used feature selection algorithms include hierarchical clustering (89), the least absolute shrinkage and selection operator regression algorithm (90) and the genetic algorithm (91). The selected discriminant features are used alone or in combination with clinical data to train the ML classifiers for breast mass classification. More recently, the potential of using DL radiomics to facilitate the classification of breast masses has also been confirmed, which was expected to identify vast arrays of quantitative features (92, 93). Zhang et al. used a CNN to extract 768 radiomic features from segmented BMUS and SWE images to further build radiomics scores, which was then confirmed to have a better performance than the radiologist assessment using BI-RADS and quantitative SWE features for discriminating benign from malignant breast masses (92). However, this study has the limitation of complicated segmentation tasks. Zhou et al. utilized a CNN for both radiomics feature extraction and breast masses classification, and 4224 low-level and high-abstract features were extracted directly from 540 SWE images that does not need object segmentation (93). The model reached a classification accuracy of 95.8%, a sensitivity of 96.2% and a specificity of 95.7%.

Since TL is an effective strategy to augment accuracy and to reduce training time by transferring the knowledge learned from a source domain to a target domain, Fei et al. proposed a projective model-based multilayer kernel extreme learning machine to transfer parameters (94). The SE-based diagnosis with BMUS imaging used as the source domain generated the best performance, with an accuracy, sensitivity, and specificity of 87.12, 86.06, and 88.15, respectively. Liao et al. applied the VGG-19 network pretrained on the ImageNet dataset based on either single-modal or multi-modal images for breast tumor classification (95). The combination feature model based on BMUS and SE images yielded a correct recognition rate of 92.95% and an AUC of 0.98.

### Prediction of ALN status and treatment response

Accurate preoperative evaluation of ALN status in patients with breast tumors is important for surgical decisions and prognosis (96). To analyze ALN status, several AI models have been proposed based on the ultrasound images of ALNs (97) or of primary breast tumors (98, 99). For the differentiation of disease-free axilla and any axillary metastasis in early-stage breast cancer, Our team (98) and Zheng et al. (99) proposed to combine the radiomics features from BMUS and SWE with independent clinical risk factors, all generating satisfactory results. In addition, the DL radiomics model of Zheng et al. could also discriminate between a low and heavy metastatic axillary nodal burden, with an AUC of 0.905 (99).

Knowing how the tumor responds to treatment can be helpful for subsequent treatment selections. Furthermore, it is important to evaluate the pathological complete response (pCR) in breast cancer, since it is related to the rates of long-term survival. Fernandes et al. found that the SR could be predictive of the neoadjuvant chemotherapy response in locally advanced breast cancer (100). A significant difference in tumor stiffness was observed as early as 2 weeks into treatment. By using the preoperative data, the Naïve Bayes classifier achieved a classification of pCR and npCR with a sensitivity of 84%, a specificity of 85%, and AUC of 81%. The detailed results are summarized in **Table 3**.

**Table 3:** The performance summary of artificial intelligence applied to ultrasound elastography in the evaluation of breast masses.

Year	Authors	No. of patients	Device	Modality	Training targets	Method		Features		Performance
2008	Nedevschi et al. (69)	30	NA	SE	Segmentation	DAEM algorithm		Quantitative features		Error of 5%
2009	Moon et al. (78)	181	Hitachi	BMUS, SE	Classification	ANN		Quantitative features		AUC: 0.92 Accuracy: 90.6% Sensitivity: 95.6% Specificity: 87.6%
2012	Selvan et al. (79)	40	Siemens	BMUS, SE	Segmentation, Classification	LSM, SRAD, FLS		Texture features, quantitative features		Accuracy: 83% Sensitivity: 100% Specificity: 74%
2014	Lo et al. (71)	112	Siemens	BMUS, SWE	Segmentation, Classification	LSM, LR		Quantitative features		AUC: 0.90 Accuracy: 84% Sensitivity: 80% Specificity: 87% (The combined features)
2014	Xiao et al. (74)	125	SuperSonic	SWE	Segmentation, Classification	M-S model, LSM, SVM		Quantitative features		AUC: 0.97 Accuracy: 95.2% Sensitivity: 90.9% Specificity: 97.5%
2015	Lo et al. (72)	90	Siemens	BMUS, SE	Segmentation, Classification	LSM, FCM clustering		Quantitative features		AUC: 0.93 Accuracy: 86% Sensitivity: 87% Specificity: 84% (The combined features)
2015	Zhang et al. (76)	125	SuperSonic	SWE	Segmentation, Classification	Fisher classifier		Texture features		AUC: 0.968 Accuracy: 92.5% Sensitivity: 89.1% Specificity: 94.3% (The feature T_mean_)
2015	Selvan et al. (80)	62	NA	BMUS, SE	Segmentation, Classification	LSM, SRAD, BPN		Texture features, quantitative features,		Accuracy: 82.3% Sensitivity: 92.9% Specificity: 73.5% (The combined features)
2015	Ara et al. (81)	170	Ultrasonix	BMUS, SE	Classification	GA, Linear classifier		Quantitative features		Accuracy: 96.5%-99.4% Sensitivity: 94.6%-98.2% Specificity: 97.2%-100.0% (The bimodal index)
2016	Zhang et al. (85)	121	SuperSonic	SWE	Classification	PGBM, RBM, SVM				AUC: 0.947 Accuracy: 93.4% Sensitivity: 88.6% Specificity: 97.1%
2017	Moon et al. (75)	109	SuperSonic	BMUS, SWE	Segmentation, Classification	LSM, SVM		Quantitative features		AUC: 0.96 Accuracy: 92.3% Sensitivity: 90.4% Specificity: 94.7% (The combined features)
2017	Zhang et al. (89)	117	Hitachi	SE	Classification	Chan-Vese level sets, morphologic closing operation, contourlet transformation, hierarchical clustering, SVM		Radiomic features		AUC: 0.917 Accuracy: 88.0% Sensitivity: 85.7% Specificity: 89.3%
2017	Zhang et al. (97)	158	Esaote	BMUS, SE	Predict ALN status	SVM		Quantitative features		AUC: 0.895 Accuracy: 85.7% Sensitivity: 84.8% Specificity: 87.0% (The combined features)
2018	Marcomini et al. (73)	83	Canon	SE	Classification	NA		Quantitative features		AUC: 0.853 Sensitivity: 71.0% Specificity: 88.5%
2018	Fleury et al. (82)	83	Canon	BMUS, SE	Classification	NA		Quantitative features		AUC: 0.868-0.926 Accuracy: 71.1%-77.1% Sensitivity: 96.8%-100% Specificity: 55.8%-63.5% (USdx combined with CADxSE software)
2018	Yu et al. (84)	187	SuperSonic	SWE	Segmentation, Classification	Wavelet transformation, LSM, SVM		Texture features, quantitative features		Accuracy: 94.8% Sensitivity: 95.1% Specificity: 94.6%
2018	Zhou et al. (93)	205	SuperSonic	SWE	Classification	CNNs		Radiomic features		Accuracy: 95.8% Sensitivity: 96.2% Specificity:95.7%
2019	Sasikala et al. (77)	113	Epiq	BMUS, SE	Segmentation, Classification	SRAD, PSO, FLS, SVM		Texture features		Accuracy: 96.2% Sensitivity: 94.4% Specificity: 97.4% (Feature LBP)
2019	Zhang et al. (88)	121	SuperSonic	BMUS, SWE	Classification	RD-GAD, DPN				AUC: 0.961 Accuracy: 95.6% Sensitivity: 97.8% Specificity: 94.1% Youden’s index: 91.9%
2019	Fei et al. (94)	264	Mindray	BMUS, SE	Classification	ML-KELM-PM				Accuracy: 87.12% Sensitivity: 86.06% Specificity: 88.15%
2019	Fernandes et al. (100)	92	Ultrasonix	SE	Predict pCR	NBC		Quantitative features		AUC: 0.81 Sensitivity: 84% Specificity: 85%
2020	Destrempes et al. (83)	103	GE, Canon, SuperSonic	BMUS, SWE	Classification	RF		Quantitative features		AUC: 0.97 Sensitivity: 98% Specificity: 75.9%
2020	Youk et al. (90)	328	SuperSonic	BMUS, SWE	Classification	Wavelet transformations, LASSO		Radiomic features		AUC: 0.992
2020	Li et al. (91)	178	Mindray	BMUS, SWE, CEUS	Classification	Attribute bagging, GA, SVM		Radiomic features		AUC: 0.919 Accuracy: 84.12% Sensitivity: 92.86% Specificity: 78.80%
2020	Zhang et al. (92)	291	SuperSonic	BMUS, SWE	Classification	LASSO, CNNs		Radiomic features		AUCs: 0.99 in training cohort; 1.00 in validation cohort
2020	Fujioka1 et al. (86)	363	Canon	SWE	Classification	CNNs				the mean AUC: 0.870
2020	Liao et al. (95)	141	Hitachi	BMUS, SE	Segmentation, Classification	CNNs				AUC: 0.98 Accuracy: 92.95% Sensitivity: 91.39% Specificity: 94.71%
2020	Zheng et al. (99)	584	Siemens	BMUS, SWE	Predict ALN status	DLR, SVM		Radiomics features		ALN status between N0 and N_+_(≥1): AUC: 0.902 ALN status between N_+_ (1, 2) and N_+_(≥3): AUC: 0.905
2021	Li et al. (87)	91	SuperSonic	BMUS, SWE	Classification	CNNs				AUC: 0.892 Accuracy: 92.4% Sensitivity: 81.5% Specificity: 96.9%
2022	Jiang et al. (98)	433	SuperSonic	BMUS, SWE	Predict ALN status	MRMR, LASSO, LR		Radiomic features		Overall C-index: 0.842 in the training set

ALN, axillary lymph node; ANN, artificial neural network; BMUS, B-mode ultrasound; CADxSE, a CAD system for analyzing SE; CEUS, contrast-enhanced ultrasound; CNN, convolutional neural network; DAEM, Deterministic Annealing Expectation Maximization; DLR, deep learning radiomics; DPN, deep polynomial network; FCM clustering, fuzzy c-means clustering; FLS, fuzzy logic system; GA, genetic algorithm; LASSO, least absolute shrinkage and selection operator; LSM, level set method; LR, logistic regression; ML-KELM-PM, a projective model based multilayer kernel extreme learning machine algorithm; MRMR, minimum redundancy maximum relevance; M-S model, Mumford-Shah function; NA, not available; NBC, Naïve Bayes classifier; pCR, pathological complete response; PGBM, point-wise gated Boltzmann machine; PSO, Particle Swarm Optimization; RBM, restricted Boltzmann machine; RD-GAD, the reaction diffusion level set model combined with the Gabor-based anisotropic diffusion algorithm; RF, random forest; SE, strain elastography; SRAD, Speckle Reducing Anisotropic Diffusion; SVM, support vector machine; SWE, shear wave elastography; USdx, BI-RADS ultrasound lexicon.

## Thyroid nodule evaluation

Thyroid nodular diseases are very common, and the incidence of thyroid cancer has increased worldwide year by year (101). However, only 5%-15% of thyroid nodules are malignant and the majority of thyroid nodules selected for fine-needle aspiration biopsy are benign (102, 103). The current clinical challenge is to discriminate the few clinically significant malignant nodules from the many benign nodules and thus identify patients who warrant surgical excision, hence to decrease medical costs and patient suffering. Moreover, lymph node (LN) metastasis is highly associated with local recurrence, distant metastasis and thyroid cancer staging, which will further guide the surgical plan. Therefore, a credible and noninvasive method is highly desirable for evaluation of thyroid nodules, including classification of thyroid nodules or prediction of the lymph node metastasis.

### Segmentation and delineation of thyroid nodules

Segmentation plays an essential role in AI-based USE models, for that malignant thyroid nodules can be accurately diagnosed by using features of well-segmented nodules. However, USE images often have low image quality due to the existing high noise, making automated segmentation a tough task. To segment the thyroid nodules in a noisy environment, Huang et al. proposed a new segmentation method based on the adaptive fast generalized fuzzy clustering algorithm by utilizing the gray level and spatial position information of the original image (104). The proposed method obtained segmentation accuracies of 0.9981 in Gauss noise (0.03) and 0.9986 in Gauss noise (0.05), indicating that it had a strong ability to suppress noise and obtained more accurate results when clustering images with high noise.

### Classification and diagnosis of thyroid nodules

Although the indices such as ES and SE have been introduced, SE is still a quantitative and subjective imaging method. Ding et al. computed a quantitative metric “hard area ratio” by transferring the original color thyroid elastograms from the red–green-blue color space to the hue-saturation-value color space (105). The SVM classifier obtained an accuracy of 93.6% when the hard area ratio and textural feature were used. Two studies used logistic regression analysis to investigate which sonographic features were associated with the malignancy of thyroid nodules and established formulas for predicting whether thyroid nodules were malignant or benign (106, 107).

Whether the ML-based diagnostic pattern can provide a more effective and accurate diagnosis than human experts for thyroid nodule classification still remains unknown. In a large study including 2064 thyroid nodules, Zhang et al. compared the diagnostic performance of nine ML classifiers trained on 11 BMUS features and 1 SE feature with experienced radiologists for thyroid nodule discrimination (108). The RF classifier generated the highest AUC of 0.938, performing better than radiologist diagnosis based on BMUS only (AUC= 0.924 vs. 0.834) and based on both BMUS and SE (AUC= 0.938 vs. 0.843). Recently, both ML-based visual and radiomics are popular methods used to diagnose thyroid nodules, Zhao et al. found that the ML-assisted US visual approach had the best diagnostic capability compared with radiomics approach and American College of Radiology Thyroid Imaging Reporting and Data System (ACR TI-RADS) (AUCs: 0.900 vs. 0.789 vs. 0.689 for the validation dataset, 0.917 vs. 0.770 vs. 0.681 for the test dataset) (109). When employing the ML-assisted US+SWE visual approach, the unnecessary fine-needle aspiration biopsy rate decreased from 30.0% to 4.5% for the validation dataset and from 37.7% to 4.7% for the test dataset.

Since DL is data-hungry, and the lack of standardized image data may lead to the overfitting problem, the TL strategy has also been applied to a few studies for thyroid nodule classification (110, 111). Qin et al. proposed to transferring feature parameters learned from VGG16, which was pretrained on ImageNet, to ultrasound images and used the hybrid features of BMUS and SE images to build an end-to-end CNN model (110). The proposed AI-based method yielded an accuracy of 0.9470, which was better than other single data-source methods. Pereira et al. compared the performance of conventional feature extraction-based ML approaches, fully trained CNNs, and TL-based pretrained CNNs for the detection of malignant thyroid nodules (111). The results showed that the pretrained network yielded the best classification performance with an accuracy of 0.83, which was better than that of fully trained CNNs. This may be caused by the relatively limited sample size used to train the fully trained network.

### Prediction of CLN status

Although papillary thyroid cancer (PTC) is an indolent type of cancer, 20-90% of PTC patients are diagnosed with cervical lymph node metastasis (CLNM) (112), which is highly related to recurrence and a poor survival rate. Accurate CLNM estimations of PTC patients are clinically important. Liu et al. built a radiomics-based model, which extracted 684 radiomic features from both BMUS and SE images to estimate LN metastasis for PTC patients, yielding an AUC of 0.90, which was better than using features extracted from BMUS or SE separately (113). However, it only utilized the radiomics features, with no consideration on other clinical information. Our team developed a radiomics nomogram by incorporating SWE radiomics features as well as clinicopathological risk factors for predicting CLNM in PTC patients, which showed good diagnostic performance in the training set (AUC of 0.851) and the validation set (AUC of 0.832) (114). The detailed performance of AI based on USE for the evaluation of thyroid nodules is summarized in **Table 4**.

**Table 4:** The performance summary of artificial intelligence applied to ultrasound elastography in the evaluation of thyroid nodules.

Year	Authors	No. of patients		Device	Modality	Training targets		Method	Features	Performance
2011	Ding et al. (105)	125		Hitachi	SE	Classification		SVM	Quantitative features, texture features	Accuracy: 93.6% AUC: 0.97
2016	Bhatia et al. (106)	105		SuperSonic	SWE	Classification		LR	Quantitative features, texture features	AUC: 0.973 Sensitivity: 97.5% Specificity: 90.0%
2017	Pang et al. (107)	525		Siemens	BMUS, SE, CEUS	Classification		LR	Quantitative features	AUC: 0.930 Accuracy: 87.05% Sensitivity: 83.77% Specificity: 89.56%
2018	Pereira et al. (111)	165		NA	BMUS, SWE	Classification		NBC, LR, SVM, DT, CNN, TL		Accuracies: 0.80 for conventional feature extraction, 0.75 for fully trained CNN, 0.83 for pre-trained CNN
2018	Liu et al. (113)	75		Samsung	BMUS, SE	Predict CLNM		SRC, SVM	Radiomic features	AUC: 0.90 Accuracy: 0.85 Sensitivity: 0.77 Specificity: 0.88 (SVM based on US and SE features)
2019	Zhang et al. (108)	2032		Hitachi	BMUS, SE	Classification		LR, LDA, RF, SVM, AdaBoost, KNN, Nnet, NBC, CNN	Quantitative features	AUC: 0.938 Accuracy: 85.7% Sensitivity: 89.1% Specificity: 85.3% (RF based on US and SE features)
2020	Huang et al. (104)	543		SuperSonic	SE	Segmentation		AFGC	NA	SA and CS: all above 99%
2020	Qin et al. (110)	233		Philips, Siemens, Myry	BMUS, SE	Classification		CNN, TL		AUC: 0.9877 Accuracy: 94.70% Sensitivity: 92.77% Specificity: 97.96%
2020	Jiang et al. (114)	237		SuperSonic	BMUS, SWE	Predict CLNM		MRMR, LASSO, LR	Radiomic features	AUCs: 0.851 in the training set; 0.832 in the validation set
2021	Zhao et al. (109)	822		SuperSonic	BMUS, SWE	Classification		DT, NBC, KNN, LR, SVM, RF, KNN-based bagging, XGboost, MLP, GBT	Quantitative features, radiomics features	AUC: 0.951 Accuracy: 88.8% Sensitivity: 81.7% Specificity: 92.9% (KNN-based bagging model in validation data)

AFGC, adaptive fast generalized fuzzy clustering algorithm; AdaBoost, adaptive boosting; CLNM, cervical lymph node metastasis; CS, comparison scores; DT, decision tree; GBT, gradient boosting tree; MLP, multilayer perception; NA, not available; Nnet, neural network; SA, segmentation accuracy; XGboost, extremely randomized trees.

## Others

In addition to the applications mentioned above, there are some applications of AI in USE to other organs, such as the diagnosis of prostate cancer (115, 116), the prediction of LN metastasis (117, 118) and tumor deposits (119) in rectal cancer, the prediction of lung mass density lung (120–122), the evaluation of plantar fasciitis (123), as well as the differential diagnosis of brain tumors (124, 125) or the prediction of the overall survival in glioblastomas (126).

## Discussion and conclusion

In conclusion, the diagnostic abilities will be extensively enhanced with the AI methods applied to USE images, especially to the multi-modal images, including ultrasound imaging methods (BMUS, USE and CEUS) and other medical imaging techniques (MRI and CT). Although the available studies have all revealed that the models based on multimodal images had superior performance to those based on single modality, the actual selection in the model development depends on the availability of datasets. One the one hand, an additional imaging modality can help provide more effective and comprehensive information. If the multi-modal imaging data is available and acquired standardized, USE data in combination with BMUS or other modalities is expected to improve the diagnostic accuracy. On the other hand, the model based on unimodal images yielded acceptable performance and the standardized and curated unimodal images are usually more easily-obtained. AI models based on them are also easier to use and generalize in the clinic, especially in primary hospital. Thus, higher accuracy should not be the only factor taken into account when selecting a unimodal or multimodal prediction model; the model’s applicability at various institutions should also be considered.

Overall, according to the available studies, AI technology is a powerful tool to assist different clinical tasks of different diseases with a comparable consistency. Although the diagnostic performance varies in different diseases, AI methods applied to USE imaging demonstrated remarkable capability for the differentiation of malignant or benign breast masses, focal liver lesions and thyroid nodules, the staging of liver fibrosis and the prediction of lymph node metastasis and so on. The performance of many applications has been shown to be comparable or even better than that of experienced radiologists. This might be facilitated by the increasing availability of curated datasets and the optimized AI architectures. However, further validations with extensive datasets are still needed to affirm the performance. In addition, the nonuniform acquisition methods and variability of ultrasound data are major challenges that restrict the comparison and generalization of different methods in different tasks. The construction of standard databases for different ultrasound applications is a future direction in further studies.

In general, early ML-based USE models have an emphasis on the extraction of discriminant features from the USE images alone or the combined BMUS and USE images, with texture features and elasticity indices mostly utilized. Then, these features will be input into classical ML classifiers, such as SVM, RF or ANN etc., for a more accurate and effective diagnosis. Since feature calculations and image segmentation are not required and hierarchical features can be automatically learned, some DL architectures, including CNNs and other popular DL architectures based on different training strategy such as GoogleNet, AlexNet, VGG, ResNet and DenseNet, are also increasingly being applied to mitigate the limitations of traditional ML processes. Furthermore, dozens of notable radiomics studies have been enabled since radiomics allows quantitative extraction of high-throughput features from medical images that are not directly visible to the naked eyes, and then ML methods are applied to build classification or prediction models based on the radiomics features alone or the incorporation of disease-correlated clinical information.

However, there are still some challenges to generalizing AI methods applied to USE in the clinical practice. The lack of large curated data has been considered as the one of the main challenges. Given that DL algorithms are “data hungry”, large-scale multicenter studies with well-annotated datasets are needed to further determine the diagnostic values of AI in USE imaging. The lack of a dataset will increase the risk of overfitting, which will occur when a model has too many parameters and remembers the training data but cannot be generalized to new independent data. One common solution is the data augmentation method. Data can be artificially augmented by random transformations, including flipping, rotation, translation and zooming (13). Another common strategy is the TL, which can transfer the parameters learned from one dataset to another target dataset. Recent advances of novel DL architectures, including the unsupervised learning, Generative Adversarial Networks (GANs) (127) for example, and federated learning (128) have shown great promise to circumvent the obstacle of scarce data. GANs, consisting of one generator and one classifier, can help generate synthetic medical images to train deeper architectures. GANs have been shown to be very effective at medical image synthesis between magnetic resonance imaging (MRI) and computed tomography (CT) images (129, 130). Federated learning enables multiple parties to collaboratively construct a ML model based on datasets that are distributed across multiple devices while keeping their private training data private. It can be useful to help address the problems related to privacy and ethical when patients’ data are sharing among different centers. It’s also worth mentioning the Graph Neural Networks (GNNs), which concentrates on learning the data represented in the form of graphs (131). GNNs have been applied to several computer vision tasks, including few-shot image classification (132) and image segmentation (133). Nevertheless, these advancements have not yet been explored in USE imaging and are likely to be promising approaches. The non-interpretability of DL approaches is another challenge. It is also known as the “black box”, which means that it is difficult for radiologists to explain the results given by DL architectures. Since there is a lack of understanding of the relationship between the input and output, identifying the features actually used for interpretation seems to be impossible, and radiologists may not accept the conclusions derived from such an AI architecture. The advance of heatmap may help to address this problem.

In addition to the above impediments, USE presents unique challenges. First, irrelevant patterns such as noises, artifacts and regions lacking USE information can often be detected, which will increase the difficulty for manual or automatic feature extraction. The image quality of USE still needs to be improved. Secondly, because ultrasound is often used as a first-line imaging modality, there is often an imbalance with an excess of normal or healthy images than abnormal or unhealthy ones, which will reduce the diagnostic accuracy of AI models. In addition, the generalizability of the developed AI models is another challenge. Most datasets are generated from a single device type and a single collection center, and most of the present AI has concentrated on single task within an overall system, such segmentation or classification, all limiting the generalizability of the developed AI models.

AI is unlikely to replace radiologists in the near or far future, owing to the complexity of creating and training an AI architecture. What is imperative is that radiologists should understand the basic working principles of AI and better apply it to medical image interpretation and analysis. Although a second opinion can be provided by AI based on USE models using ML or DL techniques, the final diagnosis decision should be made by the radiologists.

## Author contributions

All authors helped in writing and revising the manuscript and drafting the figures and tables. All authors contributed to the article and approved the submitted version.

## Glossary

**Table:**

ACR BI-RADS	American College of Radiology Breast Imaging Reporting and Data System (a standardized imaging reporting and data system for ultrasound diagnosis of breast nodules proposed by American College of Radiology)
ACR TI-RADS	American College of Radiology Thyroid Imaging Reporting and Data System (a standardized imaging reporting and data system for ultrasound diagnosis of thyroid nodules proposed by American College of Radiology)
AI	artificial intelligence (the theory and science of computer systems that are capable of human intelligence)
ALN	axillary lymph node (the lymph nodes growing in the axilla, which is a common site of metastasis for breast cancer)
ANNs	artificial neural networks (a computational framework with a large number of interconnected neurons or nodes, formed by different kinds of layers)
ARFI	acoustic radiation force impulse (a short-duration high-intensity acoustic “pushing”
CT	computed tomography (a method of examining body organs by scanning them with X rays and using a computer to construct a series of cross-sectional scans along a single axis)
BMUS	B-mode ultrasound (also known as two-dimensional gray-scale ultrasound, which is the basis for other ultrasound imaging techniques)
CEUS	contrast-enhanced ultrasound (a technique using a new contrast agent that can pass through pulmonary circulation, which can observe microcirculation perfusion of organs and tissues)
CLD	chronic liver diseases (a disease process of the liver that involves a process of progressive destruction and regeneration of the liver parenchyma leading to fibrosis and cirrhosis)
CLNM	cervical lymph node metastasis (the common metastatic sites of neck tumors, such as thyroid cancer)
CNN	convolutional neural network (a type of deep learning network using a specialized mathematical function called “convolutional”)
DL	deep learning (the use of neural networks with many layers stacked)
DLRE	deep learning radiomics of elastography (the DL-based radiomics analysis of ultrasound elastography)
ES	elasticity scores (a five-point scoring system applied in strain elastography)
FLLs	focal liver lesions (the focally formed liver lesions)
HCC	hepatocellular carcinoma (the most common pathological type of liver cancer)
LN	lymph node (an oval or kidney-shaped organ of the lymphatic system, distributed widely throughout the body including the armpit and stomach and linked by lymphatic vessels)
ML	machine learning (a branch of AI, which helps the creation of algorithms that are able to learn from data and make predictions, thus enabling computers to learn like humans)
MRI	magnetic resonance imaging (the use of nuclear magnetic resonance of protons to produce proton density images)
pCR	pathological complete response (the important prognostic factors for assessing long-term disease-free survival and overall survival in breast cancer patients)
PTC	papillary thyroid cancer (the most common pathological type of thyroid cancer)
RF	Random Forest (a notion of the general technique of random decision forests that are an ensemble learning method for classification, regression and other tasks)
ROI	region of interest (a selected subset of samples within a dataset identified for a particular purpose)
SE	strain elastography (a kind of ultrasound elastography using constant stress)
SR	strain ratio (a semi quantitative measurement that is calculated as the ratio between the strain in the normal reference region and the strain in the interested region)
SVM	Support Vector Machine (the supervised learning models with associated learning algorithms that analyze data used for classification and regression analysis)
SWE	shear wave elastography (a kind of ultrasound elastography using a ‘push’
SWV	shear wave velocity (the measured speed of the produced shear wave, which is commonly used to quantify the tissue elasticity)
USE	ultrasound elastography (a medical imaging modality that maps the elastic properties of soft tissue).
